# Malignant solitary fibrous tumor of the pleura slowly growing over 17 years: case report

**DOI:** 10.1186/1749-8090-9-113

**Published:** 2014-06-21

**Authors:** Hyun Woo Jeon, Soon Seog Kwon, Young-Du Kim

**Affiliations:** 1 Department of Thoracic and Cardiovascular Surgery, Bucheon St. Mary’s Hospital, College of Medicine, The Catholic University of Korea, 222 Banpo-daero, Seocho-gu, Seoul 137-701, Republic of Korea; 2 Department of Pulmonary and Allergic Medicine, Bucheon St. Mary’s Hospital, College of Medicine, The Catholic University of Korea, 222 Banpo-daero, Seocho-gu, Seoul 137-701, Republic of Korea

**Keywords:** Solitary fibrous tumor, Pleura, Thoracotomy

## Abstract

Although a solitary fibrous tumor of the pleura (SFTP) is a rare disease, and usually has a benign course, it has a malignant potential. We report a case of malignant SFTP treated surgically. A 75- year-old female was admitted with a chief complaint of hemoptysis of two weeks duration. Computed tomography of the chest imaged a large mass in the right hemithorax, which compressed adjacent organs; however, there was no evidence of invasion. We reviewed the patient’s medical records and found that the mass had been presented for 17 years. Complete resection was achieved through a right thoracotomy and histopathologic examination confirmed a malignant SFTP.

## Background

A solitary fibrous tumor of the pleura (SFTP) is a rare disease entity that usually arises from the submesothelial mesenchymal cells; its incidence is < 5% of all pleural tumors. The clinical course is usually indolent, and prognosis is favorable if complete resection is possible. However, it has been reported that 10-30% of SFTPs are malignant [1,2]. We present a case of malignant SFTP, which was successfully treated by complete resection.

## Case presentation

**Figure 1:**
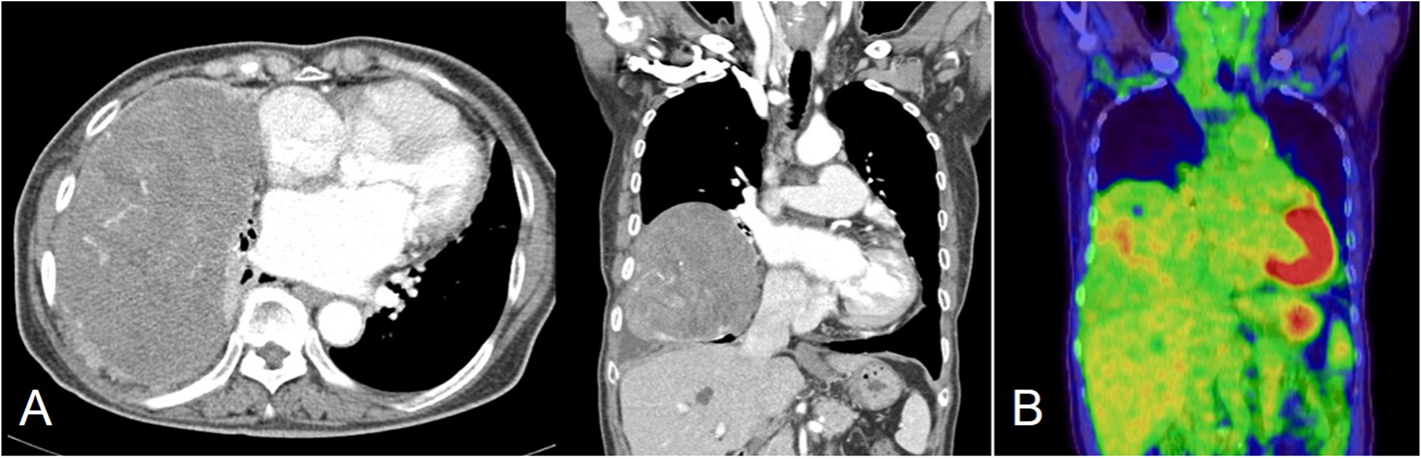
**Pre-operative imaging finding. (A)** Computed tomography of the chest revealed a large mass in the right pleural cavity, which was inhomogeneous with peripheral enhancement. **(B)** Positron emission tomography-CT also showed a pleural mass with low metabolic activity (standardized uptake value: 3.17).

**Figure 2:**
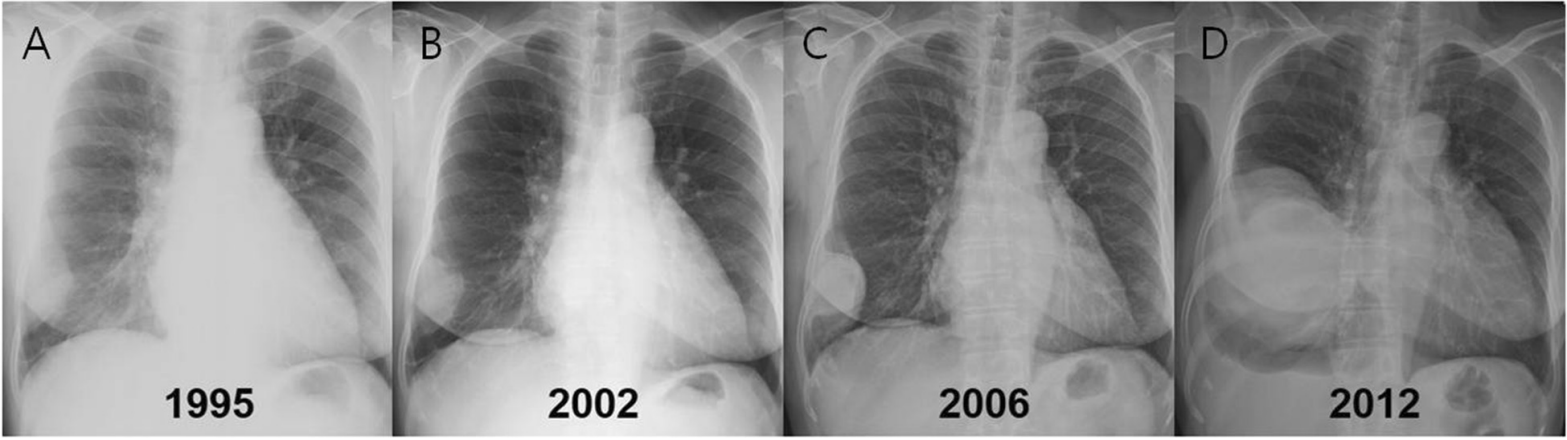
**Serial chest radiographs from 1995 through 2012. (A)** The triangular-shaped mass in the right pleural cavity was identified. **(B)** The mass was stable. **(C)** The mass exhibited progression in 2006. **(D)** The mass had grown significantly.

**Figure 3:**
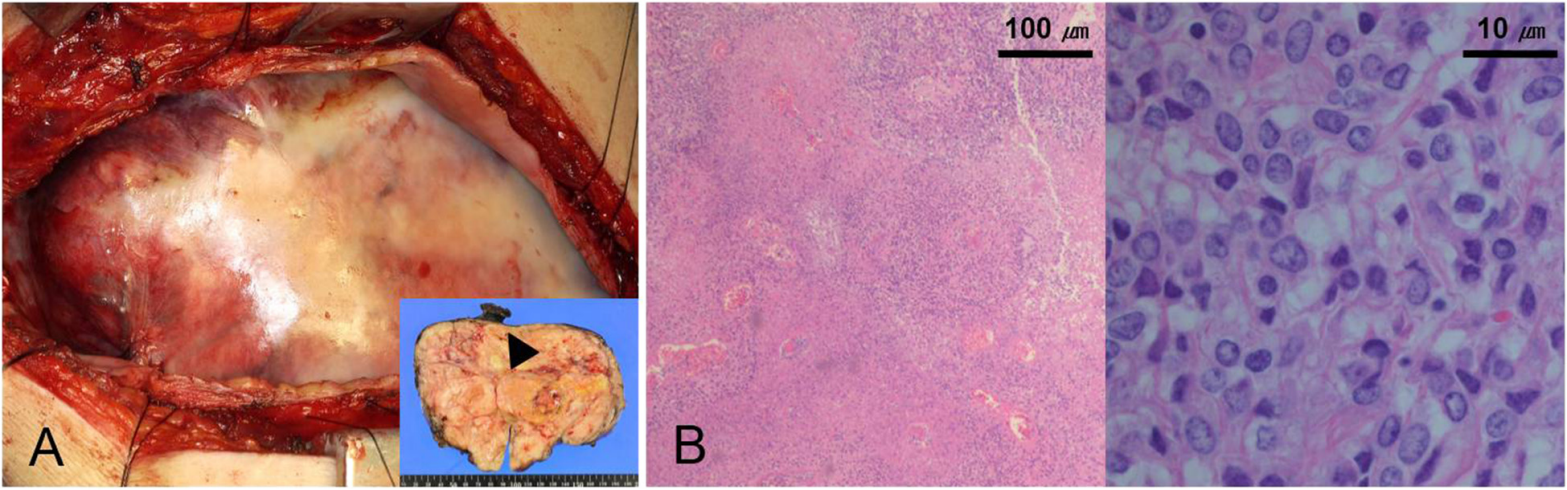
**Intraoperative and pathologic finding. (A)** Posterolateral thoracotomy revealed a large encapsulated mass measuring 16×11×7 cm that weighed 1,040 g. The mass was pedunculated and attached to the visceral pleura of the right lower lobe (arrowhead). **(B)** Microscopic findings, including marked cellularity, nuclear atypism, presence of mitosis, and necrosis, confirmed the diagnosis of a malignant SFTP.

A 75-year-old Korean female was admitted to our hospital with a chief complaint of hemoptysis of two weeks duration. She had a history of mitral stenosis and atrial fibrillation treated with medications including digoxin and warfarin; seventeen years ago she underwent right leg amputation due to femoral artery thrombosis. A computed tomographic (CT) scan of the chest revealed a large mass in the right thoracic cavity. Its internal contents were inhomogeneous; however, invasion of the chest wall or mediastinum was evident (Figure 1A). In addition, metabolic activity was low (maximum standardized uptake value: 3.7) on positron emission tomography (Figure 1B). Seventeen years ago, she was referred to our emergency room because of decreased sensorium and a history of right leg swelling with necrosis. A brain CT revealed a cerebral infarction and femoral artery thrombosis was diagnosed by an abdominal CT. Echocardiography revealed atrial fibrillation, mitral stenosis and pulmonary hypertension. Chest x-ray showed a triangular opacity in the peripheral lung (Figure 2A); this finding was considered to be due to a pulmonary infarction because of the clinical presentation of multiple thromboses and heart disease. Thus, no further evaluation was made and she underwent a right leg amputation. In 2006, she was referred to our hospital because of chest pain. Chest x-ray showed that the mass was stable (Figures 2B, 2C). A percutaneous CT-guided biopsy revealed a benign SFTP. She refused further treatment and she was lost in follow-up until 2012 when she presented with hemoptysis and a large mass with mediastinal shifting on chest x-ray (Figure 2D).Percutaneous CT-guided biopsy was conducted and histology was negative for cytokeratin, desmin, S-100 and positive for CD 34. Ki-67 was < 1%. Furthermore, mitosis, pleormorphism, necrosis and hemorrhage were absent. Although the histology was benign, we performed a right thoracotomy because of the size of mass. The mass was densely adhered to the lung, chest wall, diaphragm, and pericardium. There was no evidence of invasion of the surrounding tissue; thus, it could be resected completely, A wedge resections of the right middle and lower lobe was done; the size of the mass was 16.0 × 11.0 × 7.0 cm (Figure 3A). Histopathologic examination with the aid of immunohistochemical staining confirmed an SFTP; the tumor cells were positive for CD 34 and vimentin; however, they were negative for actin, desmin, HMB-45, CD 117, S-100, calretinin, and cytokeratin. In addition, the tumor was diagnosed as a malignant SFTP, according to the following criteria: hypercellularity, nuclear atypia, > 4 mitoses per 10 high-power fields, hemorrhage and necrosis (Figure 3B); Ki-67 was 7%. Her postoperative course was uneventful, and she has been doing well for two years after the surgery without any evidence of recurrence or metastasis.

## Discussion

SFTPs arise from the mesenchymal cells of the pleura and are usually benign; they have a good prognosis with a 10-year survival rate up to 98% [1]. The incidence is rare and is predominant in the sixth and seventh decades with no difference in gender distribution [3]. Most of the patients are asymptomatic and respiratory symptoms are the most common in symptomatic patients. Extrathoracic manifestations are also presented including osteoarthropathy and clubbing. A chest CT is the gold standard for diagnosis; however, PET CT scan has diagnostic limitations. Preoperative CT-guided biopsy is controversial because, as exhibited in our case, its accuracy rate is compromised because SFTPs involves varying cellular areas [1]. Complete resection should be performed for diagnosis and treatment. Unlike SFTPs. malignant SFTPs can have a poor prognosis, which is often associated with tumor size [4]; they can recur or metastasize, and their incidence has been reported to range from 14% to 43% and the 5-year survival rate has been reported to range from 45-68% [5,6].

Malignant SFTPs are diagnosed based on histologic finding including hypercellularity, mitosis, pleomorphism, hemorrhage and necrosis. Although some prognostic factors, such as over expression of Ki-67, extension beyond the capsule and negative conversion of CD34 have been discussed, based on the criteria of malignancy developed by England and coworkers [7]. To date, the morphological characteristics that predict a poor prognosis of SFTP have not been established [8,9]. Complete resection with lung-sparing is the treatment of choice; furthermore, malignant SFTPs can also have a favorable prognosis, if complete resection is possible [1,10].

These tumors are sometimes rapid progression [11] so these tumors should be resected.

Although the SFTP of our patient was malignant, the tumor could be completely resected, and prognostic factors other than England’s criteria were negative; therefore, a good prognosis is expected.

## Conclusions

We report a case of large malignant SFTP, which had been untreated for many years. The tumor was successfully treated by complete resection. However, long-term follow up is required, due to the potential risk of local recurrence or distant metastasis.

## Consent

Written informed consent was obtained from the patient for publication of this Case report and any accompanying images. A copy of the written consent is available for review by the Editor-in-Chief of this journal.

## Abbreviations

CT: Computed tomographic; SFTP: Solitary fibrous tumor of the pleura; PET: Positron emission tomography.

## Competing interests

The authors declare that they have no competing interests.

## Authors’ contributions

HWJ reviewed the medical record and drafted the manuscript and SSK reviewed the medical record and carried out revision. YDK reviewed the medical record of the patient and carried out operation and revision of the manuscripts. All authors read and approved the final manuscript.
